# Combined Experimental, DFT, and MD Investigation Toward the Rational Design of Desert Planting Substrates

**DOI:** 10.3390/molecules31030508

**Published:** 2026-02-02

**Authors:** Shuangnan Li, Linjie Wang, Yinghui Li, Zhenyu Zhang, Jidun Fang, Shiling Yuan

**Affiliations:** 1 School of Chemical Engineering, Shandong Institute of Petroleum and Chemical Technology, Dongying 257061, China; 2019021@sdipct.edu.cn (S.L.); thulyh@hotmail.com (Y.L.);; 2 Shandong Key Laboratory of Green Electricity & Hydrogen Science and Technology, Dongying 257061, China; 3 Institute of Carbon Neutrality, State Key Laboratory of Heavy Oil Processing, Dongying 257061, China; 4 School of Chemistry and Chemical Engineering, Shandong University, Jinan 250100, China

**Keywords:** desert ecological restoration, planting substrate, superabsorbent polymer, molecular dynamics, structure-property relationship

## Abstract

Soil moisture regulation is critical for vegetation restoration in arid ecosystems. Polymeric hydrogels, notably polyacrylic acid (PAA) and polyacrylamide (PAM), are widely employed as water-retaining agents to enhance soil water availability. However, the coupling between their distinct chemical structures and key performance metrics, particularly cycling stability and water retention kinetics in desert substrates, remains unclear. In this work, we present an integrated experimental–computational study to establish a “molecular structure–interfacial behavior–macroscopic property” framework for PAA and PAM. The results show that PAA exhibits a higher equilibrium water absorption (WAC ~242 g/g) and more stable water uptake capacity under cycling, whereas PAM displays much higher zero-shear viscosity and pronounced shear thinning with a yield plateau (~30 Pa). DFT and MD simulations trace these macroscopic disparities to their distinct electronic structures and hydration dynamics. Specifically, PAA’s strong electrostatic interactions and extended chain conformations promote a more rigid and ordered hydration shell, whereas PAM adopts a compact structure with greater chain mobility, resulting in a less ordered hydration layer. Collectively, these findings provide a structure-property framework for the scientifically grounded selection of water-retaining agents. The integrated experimental–computational methodology presented herein establishes a predictive framework for the rational design of functional materials in arid land restoration.

## 1. Introduction

Soil moisture is a primary limiting factor for vegetation establishment and ecological restoration in arid and semi-arid regions [1,2]. With accelerating land degradation and climate variability, the development of efficient, durable water-retaining materials that can increase available water in planting substrates, stabilize moisture under wet–dry cycles, and improve plant survival has become a pressing scientific and engineering need [3]. Synthetic polymeric hydrogels, especially those based on polyacrylic acid (PAA) and polyacrylamide (PAM), are widely used as water-retaining agents. They can absorb and store large amounts of water and release it slowly to surrounding soils, thereby reducing irrigation demand and increasing establishment success in degraded landscapes [4,5,6,7,8]. However, a mechanistic understanding of how the distinct chemical structures of PAA and PAM govern their cycling stability and long-term efficacy in desert planting substrates is still lacking [9].

In practical terms, the environmental performance of water-retaining agents depends not only on their equilibrium swelling capacity but also on cyclic stability, water uptake and retention kinetics, mechanical resilience under soil stresses, and water transport within a granular matrix [10]. For PAA, the abundance of carboxyl groups confers strong polarity and ionicity that favor the formation of a tightly bound hydration shell, while PAM’s amide groups and chain compactness often yield different hydration dynamics and mechanical behavior [9,11]. Understanding the inherent mechanisms and fundamental trends of these molecular-level differences, and cross-validating simulation results with experimental data, provides a mechanistic basis for interpreting and guiding the prediction of material behavior in complex environments. This is crucial for selecting or designing materials tailored to specific desert restoration or agricultural scenarios. Such mechanistic insight will facilitate the predictive screening of candidate materials and offer a scientific foundation for developing composite systems that integrate rapid water uptake, long-term retention, and mechanical durability.

Considerable experimental work has characterized the macroscopic properties of acrylate- and acrylamide-based hydrogels: FTIR and other spectroscopies identify functional groups that participate in hydration, rheological tests reveal viscoelastic spectra and yield/flow behavior, and standardized uptake/retention assays quantify water absorption (WAC) and water uptake capacity (WUC) under controlled conditions [12,13,14,15,16]. In addition, density functional theory (DFT) calculations of electronic structure and classical molecular dynamics (MD) simulations of polymer–water systems have begun to elucidate hydrogen-bonding motifs, solvation shell geometries, and chain conformational statistics that are not directly accessible by experiments [17,18].

Nonetheless, many limitations still remain in the current work. Many experimental studies report macroscopic performance but lack direct, quantitative molecular explanations for observed differences in water affinity, retention kinetics, and rheological resilience. Computational efforts are frequently limited to short chain lengths, idealized pure-water environments, or static electronic-structure descriptors, and therefore may not capture the dynamical hydrogen-bond network, long-time diffusion behavior, or mechanochemical effects relevant to wet–dry cycling and soil interactions. Few studies systematically couple DFT-derived electronic metrics (e.g., HOMO–LUMO gaps, electrostatic potential) with MD-derived hydration dynamics and with experimentally measured WAC/WUC and rheological parameters. Therefore, the absence of such a unified “molecular structure–interfacial behavior–macroscopic property” framework prevents reliable prediction of field performance and often forces material selection to remain empirical. Moreover, neglecting realistic conditions (ionic strength, soil particle contacts, mechanical shear) risks overestimating laboratory-measured advantages when materials are deployed in desert and saline–alkali soils.

To solve these problems, we constructed an integrated experimental–computational investigation to systematically reveal the mechanistic origins of distinct water-retention behavior in PAA and PAM. We synthesized and characterized PAA and PAM samples by FTIR, measured viscoelastic properties using oscillatory rheometry, and WAC, WUC, cyclic stability, and water migration in sand-column tests that mimic soil transport. Moreover, we employed DFT and extensive classical MD simulations of multi-chain polymer systems in explicit water to compute the water absorption properties and mechanisms of PAA and PAM. Finally, through combined hydrodynamic analysis and experimental validation, we characterize water transport and retention in SAP-amended soil matrices, effectively linking atomistic-scale interactions with system-level hydrological performance.

## 2. Results and Discussion

### 2.1. Fourier-Transform Infrared Spectroscopy

**Figure 1:**
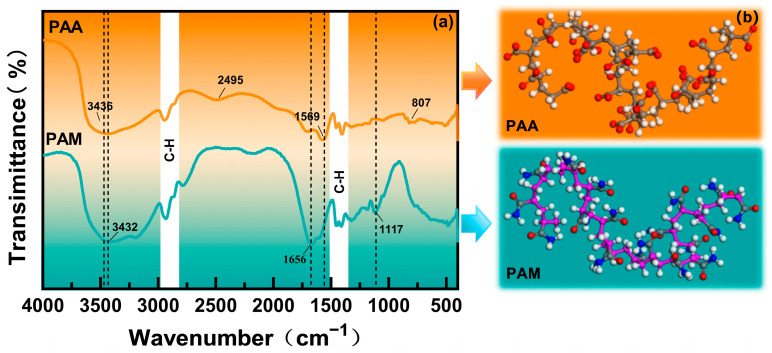
FTIR spectra (**a**) and structures (**b**) of prepared PAA and PAM.

Molecular hydrophilicity and structural stability are critical factors affecting water retention and material durability in desert composite planting substrates. The FTIR spectra of PAA and PAM are shown in Figure 1a. Both PAA and PAM exhibited a strong absorption peak at about 3400 cm^−1^. In addition, PAA showed the presence of O–H stretching vibration (3436.81 cm^−1^) and PAM exhibited that of the N–H bond (3432.25 cm^−1^). These signatures confirm the presence of polar groups (O–H/N–H) in both polymers, which are responsible for their strong hydrophilicity [19,20]. These results confirm that both polymers contain polar groups capable of forming hydrogen bonds with water, providing the chemical basis for their water-retention function in arid environments.

The distinctive peaks of PAA at 2948 cm^−1^ and PAM at 2935 cm^−1^ correspond, respectively, to the main chain skeleton. PAA had an asymmetric stretching vibration at the distinguishable peak of COO^–^ (1569 cm^−1^) [21]. PAM had distinctive absorption peaks at 1656 cm^−1^ and 1411 cm^−1^, reflecting amide I (C=O stretching) and amide II (N–H bending) vibrations, respectively. It also had a strong absorption peak near 1117 cm^−1^, which correlated to its C–N stretching vibration. As a comparison, PAA showed a weak peak near 807 cm^−1^, which was correlated to its C–O vibration. These spectroscopic assignments are consistent with and further corroborated by our subsequent molecular simulations, which reveal the expected conformational features (Figure 1b). These spectroscopic assignments, consistent with the expected molecular structures, lay the foundation for linking chemical functionality to the macroscopic hydration and mechanical properties explored in the following sections.

### 2.2. Reology

**Figure 2:**
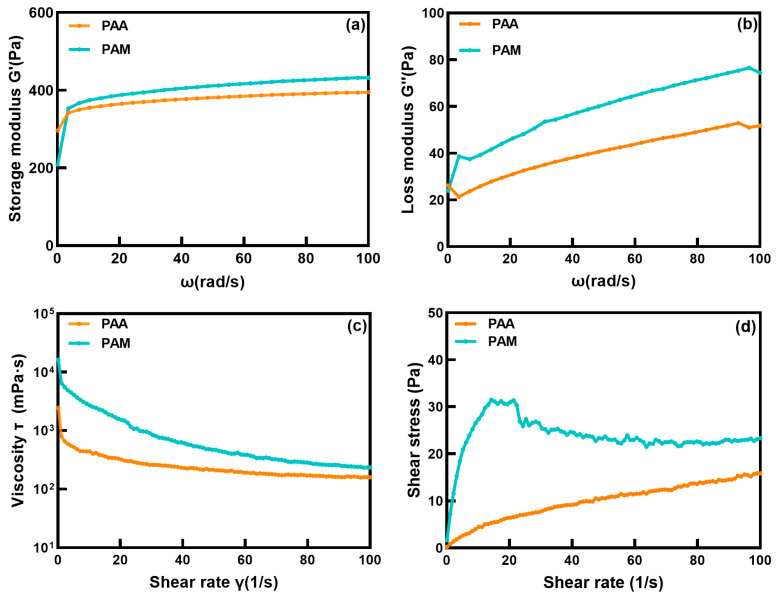
Viscoelastic behaviors of PAA and PAM: (**a**) variation in storage modulus (G′) with angular frequency (ω), (**b**) variation in storage modulus (G′′) with angular frequency (ω), (**c**) viscosity, (**d**) shear stress (τ).

To comprehensively evaluate the mechanical properties of the two water-retaining agent hydrogels and their applicability in the dynamic desert environment, we systematically conducted dynamic oscillatory frequency sweeps and steady-state shear tests. The dynamic frequency sweep results (Figure 2) clearly demonstrate that both materials exhibit typical hydrogel characteristics, with the storage modulus (G′) consistently higher than the loss modulus (G″) across the entire tested frequency range of 0.1 to 100 rad/s (Figure 2a,b). Steady-shear viscosity measurements (Figure 2c) show that both materials are shear-thinning. At a shear rate of 0.1 s^−1^, the apparent viscosity of PAM is 16,279.8 mPa·s, nearly an order of magnitude higher than that of PAA (2446.9 mPa·s), reflecting PAM’s more rigid gel network structure. As the shear rate increases to 100 s^−1^, the viscosity of PAM drops by 98.6% to 233.1 mPa·s, while PAA shows a 93.5% decrease to 158.4 mPa·s. The more drastic viscosity reduction in PAM suggests its network undergoes a more abrupt and potentially irreversible breakdown under shear, in contrast to the more gradual, likely reversible shear-thinning of PAA [22]. Steady-state shear tests further elucidate these distinct mechanical responses (Figure 2d). PAM exhibits a clear yield stress, manifested as a stress plateau at approximately 30 Pa, accompanied by a characteristic stress overshoot followed by strain-softening. This rheological signature is indicative of a brittle, solid-like network that undergoes cooperative collapse or fracture once a critical deformation threshold is exceeded [23]. In contrast, the flow curve of PAA increases monotonically without a discernible yield point or plateau. This behavior is consistent with a shear-thinning fluid, where flow proceeds through the progressive, reversible disentanglement of polymer chains, rather than through a catastrophic structural failure. Thus, the comprehensive rheological profiles delineate two contrasting mechanical identities. PAM forms a strong, elastic, yet brittle network susceptible to irreversible failure under sufficient stress. PAA constitutes a more compliant and ductile viscoelastic system capable of significant deformation and energy dissipation through reversible microstructural rearrangements. These intrinsic properties provide a foundational physical basis for understanding their potential performance in application environments involving dynamic mechanical stresses.

### 2.3. Water-Retaining Experiments

**Figure 3:**
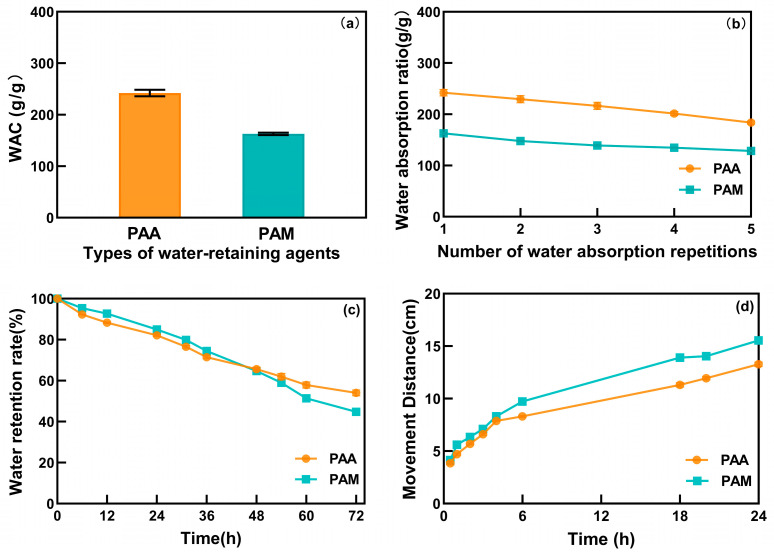
WAC and WUC comparison for PAA and PAM (**a**) water absorption multiples, (**b**) cyclic stability test in sandbox, (**c**) water retention measurement, (**d**) sand-column experiment simulating migration distance.

For the development of desert planting substrates, water-retaining agents serve as a core component for moisture regulation, and their selection must meet three key characteristics: high weathering resistance, long-term slow-release capability, and synergistic interaction with multiple components. This study systematically compares the water absorption performance, cyclic stability, and network mechanical properties of two commonly used water-retaining agents to screen matrix materials suitable for composite system construction. Experimental results demonstrate that PAA exhibits superior initial water absorption capacity, reaching 242 times its own dry mass, which is significantly higher than that of PAM, confirming its distinct advantage in rapid moisture capture (Figure 3a). However, in simulated desert wet–dry cycle tests, the water absorption performance of PAA decreased by 20% after five cycles, whereas PAM showed a reduction exceeding 25% (Figure 3b). This discrepancy suggests that although PAA undergoes slightly less degradation, both materials experience substantial performance loss under prolonged cyclic conditions.

The water uptake capacity (WUC) reflects the ability of soil to retain water over time after the application of water-retaining agents [24]. As shown in Figure 3c, the WUC of both PAA and PAM decreased over the 72 h of tests period, reaching 55.50% and 52.54%, respectively. The WUC decrease in PAM was greater than that of PAA, indicating different capabilities of sustaining water evaporation. Water transport is also important for water-retaining agent design [25]. We compared the water travel distance of PAA and PAM within 24 h, and we observed that the water transport distance, when PAA was applied, was significantly lower than that of PAM, indicating that PAA had a better water retention capability (Figure 3d). This superior retention and cyclic stability of PAA align with its flexible and reversible network structure identified in rheological analysis, which withstands repeated hydration–dehydration stresses more effectively than the brittle network of PAM.

### 2.4. Mechanistic Study Using Quantum Chemistry Calculations

**Figure 4:**
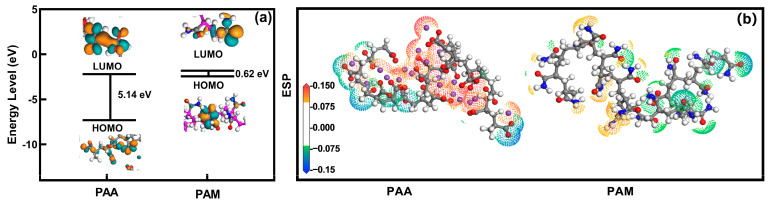
Orbital energy and electrostatic potential of PAA and PAM (**a**) HOMO and LUMO. (**b**) Electrostatic potential.

To explore active sites where the water-retaining agent binds to water molecules [26], we used the highest occupied molecular orbitals (HOMO) and the lowest unoccupied molecular orbitals (LUMO) for PAA and PAM. As shown in Figure 4a, the HOMO of PAA is located entirely on its carboxylic acid groups, while the HOMO of PAM is on its –CONH_2_ groups. The LUMO of both polymers are distributed along the polymer backbone. Orbital energy analysis further computed that the HOMO orbital energy of PAA and PAM were −7.32 eV and −2.43 eV, respectively, and the LUMO orbital energy of PAA and PAM were −2.18 eV and −1.81 eV, respectively. We quantified the orbital energy difference between HOMO and LUMO for both materials, and that difference in PAA was 5.14 eV and that of PAM was 0.62 eV. In molecular orbital theory, the HOMO–LUMO gap is an important indicator of the stability of a molecule’s electronic structure: a larger gap corresponds to a higher energy required to excite an electron from the HOMO to the LUMO, making the molecule less prone to electron transitions or participation in chemical reactions, and thus generally conferring greater chemical and kinetic stability. The significantly larger HOMO–LUMO gap of PAA (5.14 eV) compared to PAM (0.62 eV) reflects a more localized electronic structure and lower backbone polarizability for PAA [27,28,29]. This electronic rigidity is consistent with, and may partially underlie, the more extended and conformationally restricted chain architecture observed for PAA in subsequent MD simulations, which is primarily driven by electrostatic repulsion between its charged groups. However, the primary determinants of water retention and cyclic performance reside in the physical hydration structure and network dynamics, as analyzed below. 

Electrostatic potential (ESP) maps provide a direct visualization of molecular polarity and the distribution of regions prone to non-covalent interactions, such as hydrogen bonding (Figure 4b) [30]. For water-retaining agents, the magnitude and localization of the ESP are key determinants of hydration behavior. The carboxylate (–COO^−^) groups of PAA exhibit intensely negative ESP regions, signifying localized negative charge and high polarity. These strongly negative sites act as excellent hydrogen-bond acceptors, promoting the formation of strong, directional hydrogen bonds with the positively charged hydrogen atoms of water molecules. This direct link between ESP and hydrogen-bonding propensity provides a molecular-level explanation for PAA’s enhanced water affinity and high experimental water absorption capacity (WAC). In contrast, the amide groups (–CONH_2_) of PAM show a more diffuse and less negative ESP distribution. This reflects a different electronic structure with less localized charge, correlating with a relatively weaker hydrogen-bond acceptor strength and the subsequently lower WAC observed experimentally (Figure 3a) [31]. The distinct ESP features thus offer a clear electronic-structure rationale for the differing hydration capabilities and macroscopic water retention performance of PAA and PAM, complementing the dynamical insights from MD simulations.

### 2.5. Molecular Dynamics Simulation Analysis

To elucidate the influence mechanism of water-retaining agent molecular structure on their functional adaptability in the development of desert planting matrix composite systems, this study employs molecular dynamics simulation to systematically analyze the microscopic differences between PAA and PAM from the hydration structures, chain conformations, and the resulting dynamics at the polymer–water interface. The analysis focused on characterizing the order, stability, and mobility of the hydration shells, which are the key determinants of water retention capability.

**Figure 5:**
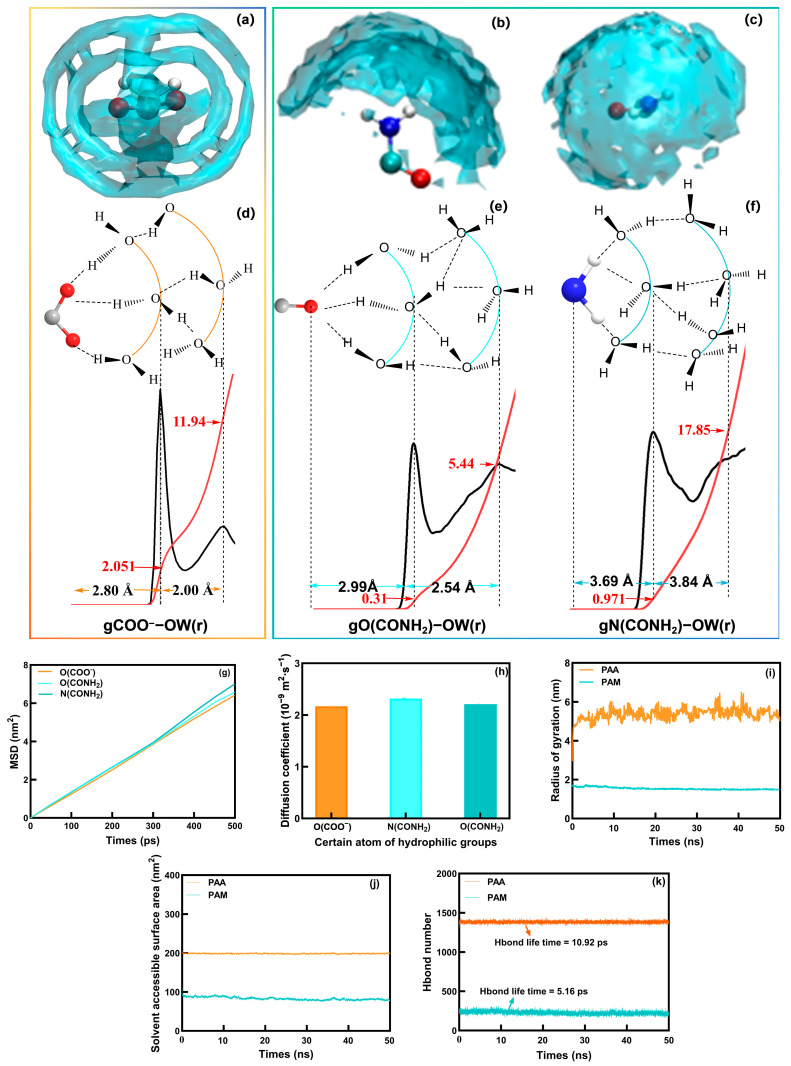
Conformation change in PAA and PAM during swelling: (**a**) spatial distribution of water around –COO^−^, (**b**,**c**) spatial distribution of water around CONH_2_, (**d**) RDF between certain atoms of hydrophilic groups and integrated hydration numbers and scheme of hydrogen bond structure in hydration shells around them gCOO^−^−OW(r), (**e**) gO(CONH_2_)−OW(r), (**f**) gN(CONH_2_)−OW(r), (**g**) MSD, (**h**) dynamic properties of water around certain atoms of hydrophilic groups, (**i**) radius of gyration, (**j**) solvent−accessible surface area, (**k**) hydrogen bond numbers.

The structural order of the hydration shell around the hydrophilic groups was first examined [32]. The spatial distribution function (SDF) in Figure 5a reveals a well-defined region of low water probability proximate to the –COO^−^ groups of PAA, indicative of a stable and ordered primary hydration shell where water molecules are tightly bound with restricted mobility [33,34]. In contrast, the SDF around PAM’s –CONH_2_ groups (Figure 5b,c) shows a more diffuse distribution, suggesting a less ordered hydration environment [35]. This qualitative observation is quantitatively supported by the radial distribution function (RDF) and the integrated hydration numbers (Figure 5d–f) [36,37]. The –COO^−^ group in PAA exhibits a sharp first peak at ~2.82 Å with a coordination number of 2.05, confirming the formation of a stable, well-defined primary hydration core where each group coordinates approximately two water molecules [38]. Conversely, the first-shell hydration numbers for the oxygen and nitrogen atoms within the PAM–CONH_2_ group are only 0.31 and 0.97, respectively, significantly lower than PAA and confirming its limited direct hydration capability. These low coordination numbers indicate a limited capacity of the amide group to directly coordinate water molecules in its primary hydration shell compared to the carboxylate group [39]. Although PAM’s amide nitrogen shows a larger second-shell coordination (17.85), when considered in conjunction with its substantially lower first-shell coordination, this likely reflects a dynamic and diffuse outer hydration layer sustained by water–water H-bonds, rather than a strongly polymer-bound structure [40]. Conversely, the second-shell hydration number for PAA−COO^−^ is 11.94. The more coherent primary and secondary hydration structure around PAA’s − COO^−^ constitutes a more stable multi-layered hydration network [41].

The structural order directly translates into pronounced dynamical restrictions on water molecules. We computed the mean square displacement (MSD) of water molecules in the first hydration layer (Figure 5g). The derived diffusion coefficients (Figure 5h) show that water around PAA’s –COO^−^ is the most severely hindered, demonstrating the strongest immobilizing effect [42]. This drastically reduced mobility at the molecular interface is consistent with the observed slower macroscopic water transport in PAA-amended sand columns (Figure 3d). Further dynamical metrics reinforce this picture. The dipole relaxation time of water molecules hydrating the –COO^−^ group (10.92 ps) is more than twice that for the –CONH_2_ group (5.16 ps), indicating a longer-lived orientation alignment and thus a more stable hydration structure in PAA. Crucially, the hydrogen-bond (H-bond) lifetime in the PAA system is also significantly longer than in PAM. These combined dynamical analyses—slower diffusion, longer dipole relaxation, and longer H-bond lifetime—collectively demonstrate that PAA creates a more persistent and “locked-in” hydration environment, which mechanistically underpins its superior water retention capacity (WUC, Figure 3c).

The stark contrast in hydration behavior is rooted in the polymers’ distinct chain conformations and their consequences for polymer–water interfacial area. As shown in Figure 5i, PAA adopts an extended conformation with a large radius of gyration (Rg ~4.5–6.5 nm), while PAM chains remain compact (Rg ~1 nm) [43,44]. This extended structure grants PAA a much larger solvent-accessible surface area (SASA ~200 nm^2^, Figure 5j) compared to PAM (~80–90 nm^2^), maximizing the contact area for hydration. Consequently, the PAA system sustains a vastly greater number of hydrogen bonds (~1400 vs. ~200 for PAM, Figure 5k), forming a robust, pervasive H-bond network that is integral to its high water uptake capacity (WAC, Figure 3a) [45,46]. The origin of these conformational differences can be attributed to their distinct chemical structures and the resulting intra- and intermolecular forces. For PAM, the neutral amide groups lack long-range electrostatic repulsion, allowing the chains to adopt compact conformations primarily driven by van der Waals interactions and backbone flexibility. This inherent conformational flexibility is consistent with its narrower HOMO−LUMO gap calculated for an isolated chain, which is often associated with a lower energy cost for electronic distortion and higher polarizability. In contrast, for PAA, the dominant factor is the strong electrostatic repulsion between the charged –COO^−^ groups, which enforces an extended and more rigid chain conformation, as reflected in its larger radius of gyration. This extended architecture is complemented by its wider HOMO−LUMO gap, indicating a more localized and stable electronic structure that is less susceptible to polarization. Thus, the DFT and MD results together point to a fundamental divergence: PAA chains are extended due to Coulombic repulsion and form a rigid hydration shell, whereas PAM chains are compact and flexible due to the absence of such repulsion, leading to a more dynamic hydration environment [47,48,49]. This difference in chain architecture—extended and electrostatically stiffened for PAA versus compact and flexible for PAM—provides the molecular basis for their contrasting macroscopic rheological behavior (PAA’s ductile shear-thinning vs. PAM’s brittle yield stress) and cyclic stability.

## 3. Materials and Methods

### 3.1. Synthesis and Processing

In this study, PAA and PAM were prepared using a solution−polymerization method. The primary instruments used in this study included: a vertical blast drying oven (Model DGG−9070B, Shanghai Senxin Experimental Instrument Co., Ltd., Shanghai, China),an electronic analytical balance (Model FA2004N, Shanghai Jingke Scientific Instrument Co., Ltd., Shanghai, China), a digital ultrasonic cleaner (Model KQ 2200 DA, Kunshan Ultrasonic Instrument Co., Ltd., Kunshan, China),a constant−temperature heating magnetic stirrer (Model DF−101S, Shanghai Lichen Bangxi Instrument Technology Co., Ltd., Shanghai, China). All chemical reagents used in this experiment were purchased from Aladdin Biochemical Technology Co., Ltd. (Shanghai, China). The specific synthesis details are provided in the Supplementary Materials. Through this synthesis route, the resulting PAA product was in the form of a partially neutralized potassium polyacrylate salt. Consequently, the majority of carboxylic acid groups in the experimental material existed in a deprotonated, anionic form (–COO^−^), with K^+^ as the counterion. In contrast, the PAM synthesized via the co−polymerization route retained neutral amide (–CONH_2_) functional groups as the primary hydrophilic units.

### 3.2. Structures and Functional Groups

Fourier−Transform Infrared Spectroscopy (FTIR) was used to characterize molecular structures of these PAA and PAM samples. The samples were dried, ground, and reshaped into pellets by mixing with KBr. The spectra were recorded in the range of 4000–400 cm^−1^ to analyze the types and relative contents of functional groups in PAA and PAM. We scanned the spectrum 15 more times to reduce noise and prevent spectrum overlapping.

Small amplitude oscillatory shear tests were also carried out using an Anton Paar MCR302e rheometer (Graz, Austria) to measure the storage modulus (G′), loss modulus (G′′), and loss factor (tanδ) of the polymers. For each measurement, 0.2 g of sample powder was weighed and mixed with 50 mL of distilled water. The viscoelastic properties were then measured at 25 °C, either at a fixed oscillation strain of 0.1% with a varying angular frequency ω from 0.1 to 100 rad/s, or otherwise at a constant angular frequency ω of 10 rad/s but with a strain amplitude ranging from 0.1% to 100%.

### 3.3. Water-Retaining Agent Benchmarking

This study systematically evaluated the application effectiveness of water-retaining agents in desert environments through tests including water absorption capacity (WAC), water uptake capacity (WUC), hysteresis and water cycling assessment, and water migration tests, with detailed experimental procedures provided in the Supplementary Materials [50].

### 3.4. Molecular Simulation Setup

We performed molecular simulations using BIOVIA Materials Studio v2025 software, employing single polymer chains consisting of 20 repeat units (20−mers) for both PAA and PAM. These geometry−optimized models were computed with the generalized gradient approximation with Perdew–Burke–Ernzerhof functional. A DFT model incorporating Becke’s hybrid functional with Lee, Yang, and Parr correlation was used to compute the electron cloud distribution and frontier orbitals of the hydrogel molecules [51]. We also employed GROMACS 2022.6 software package, using GROMOS 54a7 force field, to simulate and analyze the PAA and PAM molecular systems [52,53,54,55]. Each simulation system was constructed within a cubic periodic box of dimensions 8.0 × 8.0 × 8.0 nm^3^, containing 10 polymer chains and approximately 32,000 water molecules. To maintain charge neutrality in the PAA system, 200 K^+^ counterions were introduced to balance the fully deprotonated carboxylate groups, whereas the PAM system, being electrically neutral, required no additional ions. The resulting equilibrated structures (Supplementary Figure S5) illustrate the spatial organization of molecular components in aqueous solution. No covalent bond formation between polymer chains was observed. Instead, molecular assembly is mediated by non−covalent interactions, including electrostatic forces, hydrogen bonding, and van der Waals contacts. These simulations were carried out under conditions corresponding to pH = 7, a value representative of typical soil environments in arid and agricultural settings, thereby aligning the computational model with the intended practical application context. The dynamical trajectories were post−processed and visualized with VMD 1.9.4 software [56]. The LINCS method was implemented to constrain the bond length [57]. A cut−off radius of 1.2 nm was chosen for non−bonded interactions, and long−range electrostatic interactions were calculated using the Particle Mesh Ewald algorithm [58,59]. Water was examined using the simple point charge extended model, where periodic boundary conditions were employed to improve simulation accuracy. The temperature was controlled at 298.15 K using a V−rescale thermostat. A time step of 1 fs was used, and the trajectory information was recorded every 5 ps [60].

## 4. Conclusions

This study comprehensively evaluated two water−retaining agents—polyacrylic acid (PAA) and polyacrylamide (PAM)—by integrating macroscopic performance tests, rheological analysis, and multiscale molecular simulations. This study provides a comprehensive, multi−scale evaluation of two prevalent water-retaining agents, PAA and PAM, linking their macroscopic performance to underlying molecular mechanisms. Macroscopic performance analysis reveals that PAA exhibits superior equilibrium water absorption and cyclic stability, whereas PAM demonstrates distinct rheological behavior with a yield stress plateau, indicative of a rigid yet brittle network structure. Integrated computational analysis elucidates the molecular origins of these divergences. At the electronic structure level, DFT calculations reveal a larger HOMO−LUMO gap for PAA, which is consistent with greater electronic stability and lower backbone polarizability in the isolated state. Furthermore, MD simulations quantify that PAA forms a dense, long−lived hydrogen bond network with a larger solvent−accessible surface area (SASA) and radius of gyration (Rg). These features foster a more robust and persistent hydrogen−bond network with water, leading to significantly reduced mobility of water within the hydration layer. This mechanistically explains PAA’s excellent static water retention (high WAC) and slower water release rate (as seen in sand−column tests). In contrast, PAM exhibits fewer hydrogen bonds with shorter lifetimes and compact chain conformations. This molecular arrangement results in a less persistent hydration shell and greater water mobility, correlating with its more pronounced shear−thinning and lower water retention capacity. Ultimately, the flexible and reversible network of PAA, sustained by its extended chains and stable hydration shell, offers better durability under wet–dry cycles compared to the more brittle, entanglement−driven network of PAM. Therefore, PAA represents a more suitable candidate for desert planting applications demanding long−term durability, and the framework presented here provides a robust tool for future material design.

## Data Availability

The original contributions presented in this study are included in the article/Supplementary Material. Further inquiries can be directed to the corresponding author.

## Supplementary Materials

The following supporting information can be downloaded at https://www.mdpi.com/article/10.3390/molecules31030508/s1: Detailed material preparation procedures and experimental measurement methods for several water-retaining properties. S1: Synthesis of PAA; Figure S1: Synthesis process for PAA material used in this study; S2: Synthesis of PAM; Figure S2: Flow chart for the synthesis of PAM; S3: Water-retaining agent benchmarking; Figure S3: Comparison of PAA and PAM before and after water absorption and swelling; S4: Water Migration Test; Figure S4: Water migration test apparatus; S5: Characterization of Equilibrium Structures from MD Simulations; Figure S5: Final equilibrated structures from molecular dynamics simulations. (a) Snapshot of the polyacrylic acid (PAA) system in aqueous solution; (b) Snapshot of the polyacrylamide (PAM) system in aqueous solution.
