# Supplementary material for: Combined Experimental, DFT, and MD Investigation Toward the Rational Design of Desert Planting Substrates
Source: Molecules. 2026 Feb 2;31(3):508. doi: 10.3390/molecules31030508 (PMC12899117; doi:10.3390/molecules31030508)
Supplement: Supplementary file 1 [file molecules-31-00508-s001.zip › molecules-4090561-supplementary.pdf]

Combined Experimental, DFT, and MD Investigation Toward the Rational Design of Desert Planting Substrates

Shuangnan Li[1,2], Linjie Wang[1], Yinghui Li[1,4], Zhenyu Zhang[3], Jidun Fang[1] and Shiling Yuan[1,3*]

S1 Synthesis for PAA

We prepared the PAA polymer using the following protocol: (1) We dissolved 11.45 g of potassium hydroxide (KOH) in 25 mL of distilled water. We added 20 mL of acrylic acid, drop by drop, to the KOH solution. The mixture was kept in an ice-water bath to synthesize potassium acrylate. (2) The potassium acrylate solution was transferred into a three-necked flask, which was connected to nitrogen below the liquid surface using a gas tube and pre-purged the flask with nitrogen for 5 minutes. (3) The flask was immersed in a water bath with an initial temperature of 35°C. Potassium persulfate (0.105 g), N,N'-methylenebisacrylamide (0.012 g), and 25 mL of distilled water were added, in that order, while stirring. The mixture was gradually heated to 80°C and maintain at that temperature for 1.5 hours for polymerization. (4) After the reaction was over, the product was dried in an oven at 80°C until weight did not decline. The dried product was grounded and sieved through a 200-mesh sieve. The flowchart for this process is shown in Figure S1.

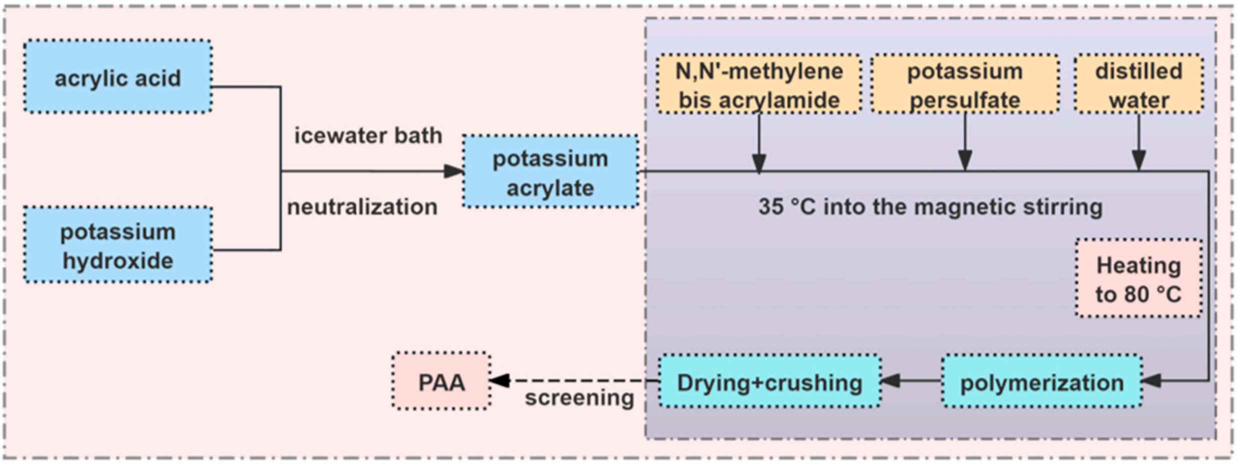

Figure S1. Synthesis process for PAA material used in this study

S2 Synthesis for PAM

The specific procedure for synthesizing PAM is as follows: (1) We dissolved 6.55 g of KOH in 30 mL of distilled water. Additional 10 mL of acrylic acid was added, drop by drop, to that solution. The mixture was then chilled in an ice-water bath to prepare potassium acrylate. (2) We used the same step as for the PAA solution above. (3) The flask was immersed in a water bath with an initial temperature of 35°C. While stirring, we added acrylamide (3.4 g), potassium persulfate (0.07 g), N,N'-methylenebisacrylamide (0.007 g), and 25 mL of distilled water in that order. The mixture was heated to 70°C and maintain at that temperature for 1.5 hours. (4) We followed the same procedure as for PAA above. The finished amorphous white powder of both PAA and PAM were kept in separate sealed containers for further assessment and experiments. The process was summarized in Figure S2.

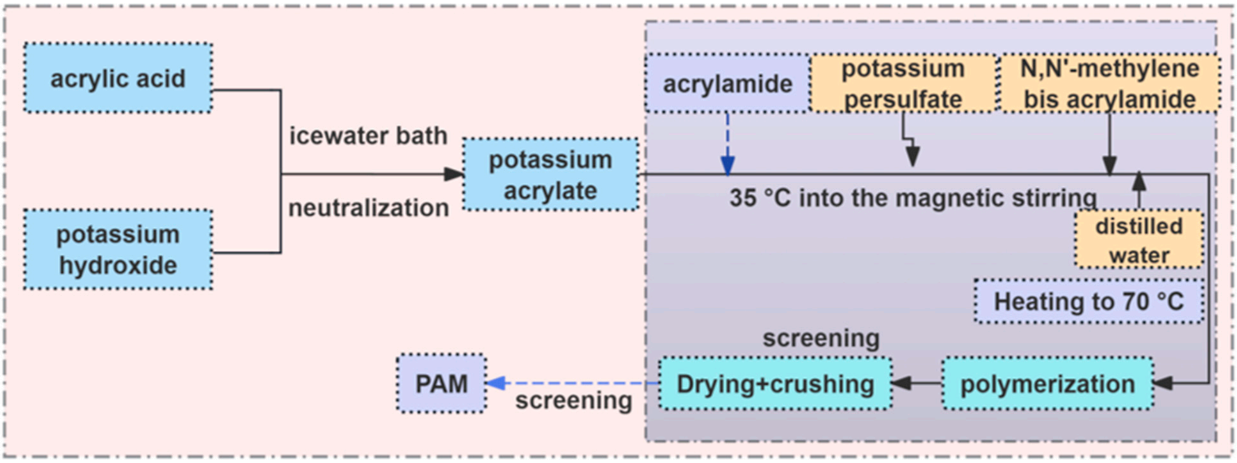

Figure S2. Flow chart for the synthesis of PAM

S3 Water Retaining Agent Benchmarking

Mass Water Absorption Capacity (WACw) is defined as the ratio of the weight of water absorbed by a material in the saturated state over the weight of the dry material at a reference condition, i.e., ambient temperature and pressure.

$$WAC_w = \frac{m_3 - m_2 - m_1}{m_1} \qquad \text{Eq. S1}$$

In this equation, WAC_w is mass water absorbing capacity (g/g);

m_3 is the mass of material saturated with water, packaged in a filter bag (g);

m_2 is the mass of the filter bag (g);

m_1 is the mass of material in absolute dry state (g).

Water Uptake Capacity (WUC) is evaluated by immersing bagged samples in water and recording its weight as time elapsed ($m_{t(t)}$), i.e., every 2hrs. WUC was calculated using use the following formula:

$$WUC = \frac{m_{t(t)} - m_1}{m_3 - m_1} \times 100\% \qquad \text{Eq. S2}$$

Water Absorption Capacity Measurements: We measured and examined WAC using the following protocol: (1) polymer powder, dried at 80°C for 48 hours, was first sampled at 0.2 gram (m_1); (2) each sample was transferred into a pre-weighed 200–mesh filter bag (m_2); (3) the filter bag was then immersed in 300 mL of distilled water in a beaker, stirred thoroughly, and settled to absorb water at the room temperature for 2 hours until equilibrium; (4) the filter bag was carefully removed, excess water was drained, and the sample was measured to obtain its fully saturated mass weight m_3; (5) WAC was finally calculated using Eq.S1. The photograph of PAA and PAM samples before and after WAC measurements is shown in Figure S3.

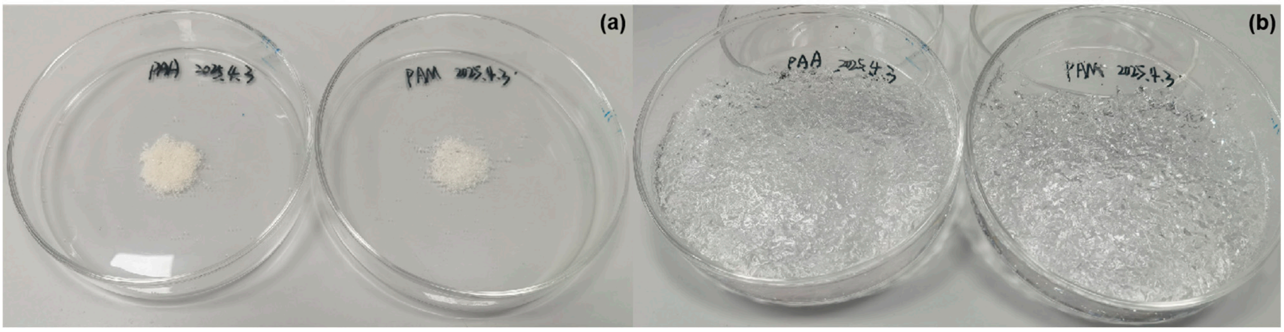

Figure S3. Comparison of PAA and PAM before and after water absorption and swelling

Hysteresis and Water Cycling Assessment: We further repeated the above-mentioned protocol for four additional times, and each time we recorded m_1, m_2, m_3, and WAC, respectively, to examine how polymer WAC might deteriorate through multiple absorption-desorption cycles, owing to hysteresis phenomena.

S4 Water Migration Test

Water Migration Test was carried out using a 20 cm diameter and 50 cm tall acrylic column, which was filled with 5 kg of dry sandy loam. Water-retaining agent, weighted at 0.5g, was mixed into the top 5 cm of the sand pack and 300 mL of water was added. The water penetration distance was measured since water addition. The experimental device and measurement is photoed in Figure S4.

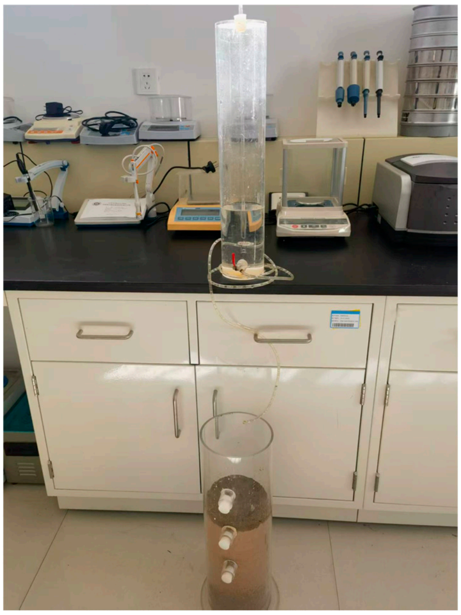

Figure S4. Water migration test apparatus

S5 Characterization of Equilibrium Structures from MD Simulations

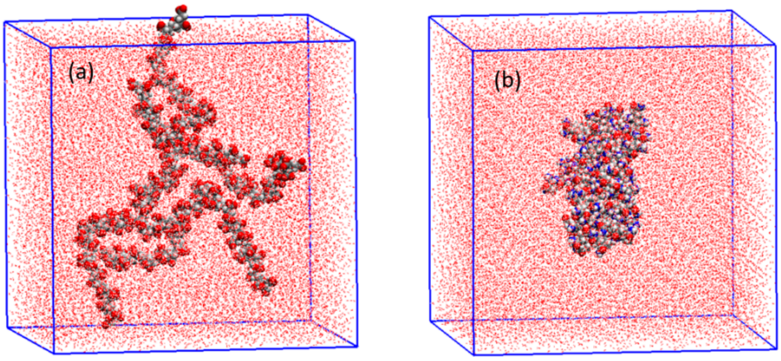

Figure S5. Final equilibrated structures from molecular dynamics simulations.(a) Snapshot of the polyacrylic acid (PAA) system in aqueous solution;(b) Snapshot of the polyacrylamide (PAM) system in aqueous solution.
